# An apoptosis-driven ‘onco-regenerative niche’: roles of tumour-associated macrophages and extracellular vesicles

**DOI:** 10.1098/rstb.2017.0003

**Published:** 2017-11-20

**Authors:** Christopher D. Gregory, Margaret Paterson

**Affiliations:** MRC Centre for Inflammation Research, University of Edinburgh College of Medicine and Veterinary Medicine, Queen's Medical Research Institute, 47 Little France Crescent, Edinburgh EH16 4TJ, UK

**Keywords:** apoptosis, cancer, repair, regeneration, macrophage, lymphoma

## Abstract

The cell-death programme, apoptosis, is well established as a tumour suppressor mechanism. Paradoxically, high levels of apoptosis in tumours are closely coupled with poor prognosis. Indeed, where it has been studied, cell loss is a striking feature of high-grade cancers, illustrating the importance of considering malignant disease as an imbalance between cell gain and cell loss that favours cell gain rather than as a unidirectional disorder of cell gain alone. In addition to orchestrating cell loss, apoptosis can signal regenerative responses—for example compensatory proliferation—in neighbouring cells. Accumulating evidence suggests that normal tissue repair and regenerative processes are hijacked in the malignant tissue microenvironment such that cancer may be likened to a ‘wound that fails to stop repairing’. We have proposed that a critical requirement for the successful growth, progression and re-growth of malignant tumours is a complex milieu, conceptually termed the ‘onco-regenerative niche’, which is composed, in addition to transformed neoplastic cells, of a network of normal cells and factors activated as if in tissue repair and regeneration. Our work is based around the hypothesis that tumour cell apoptosis, macrophage activation and endothelial activation are key, interlinked elements of the onco-regenerative niche and that apoptotic tumour cell–derived extracellular vesicles provide critical intercellular communication vehicles of the niche. In aggressive B-cell lymphoma, tumour cell apoptosis promotes both angiogenesis and the accumulation of pro-tumour macrophages in the lymphoma microenvironment. Furthermore, apoptotic lymphoma-derived extracellular vesicles have potent pro-tumour potential. These findings have important implications for the roles of apoptosis in regulation of malignant diseases and for the efficacy of apoptosis-inducing anti-cancer therapies.

This article is part of the discussion meeting issue ‘Extracellular vesicles and the tumour microenvironment’.

## 1. Introduction: apoptosis and the tumour microenvironment

Apoptosis, the most widely studied cell death programme, serves many functions in normal physiological processes, ranging from organ sculpting during embryogenesis to adult tissue homeostasis. In disease, perhaps its highest renown is as a tumour suppressor: to remove cells carrying genetic aberrations including those with oncogenic potential. In this respect, the capacity to avoid apoptosis has long been accepted as an important acquired characteristic of malignant and pre-malignant cells [1]. Consequently, anti-apoptotic pathways have been identified as important routes to malignant disease. Indeed, the prototypic anti-apoptotic gene, *BCL-2*, provided the first example of a new class of oncogenes that promote cell survival rather than cell proliferation [2]. Furthermore, apoptosis induction in tumour cells is a common mode of action both of pharmaceutical and of radiation-based anti-cancer therapies [3,4]. This demonstrates that tumour cells can retain the capacity to undergo apoptosis, a capability which, as we will discuss, lends itself to oncogenic, as well as anti-oncogenic, processes.

While it is intuitive that cell death constrains cancer, which is largely viewed as a range of diseases in which unconstrained proliferation occurs, certain characteristics of malignant disease are perhaps counterintuitive, at least when considered superficially. Firstly, cell loss is a substantial component of aggressive malignancies. Thus, where constituent cell population growth kinetics have been compared to the volumetric growth of tumours, it has been concluded that massive cell loss commonly occurs in diverse tumour types, largely through cell death [5,6]. Second, high proliferation indices are commonly accompanied by high apoptosis indices in aggressive tumours of various types [6–14]. High apoptosis indices are also reflected by high incidences of activated effector caspases—cysteinyl proteases that execute the cell death programme—in multiple malignancies (see, for example [15–19]). Strong coupling between rapid proliferation and apoptosis is common in both normal and malignant tissues. Amidst other factors, this is likely to stem not only from competition for limited nutrients but also from proliferating cells being ‘primed’ for apoptosis as described seminally as a consequence of the activity of the proto-oncogene product c-Myc [20] which is an obligate driver of both proliferation and apoptosis [21]. The intrinsic, mitochondrial pathway of apoptosis is activated in response to stress signals, including nutrient stress, chemotherapy and radiation. The critical point of no return in this pathway is mitochondrial outer membrane permeability (MOMP), which allows cytochrome *c* to be released into the cytosol to form a crucial component of the apoptosis-initiating protein complex known as the apoptosome [22]. MOMP is induced by pro-apoptotic Bcl-2 family members, Bax and Bak, and inhibited by anti-apoptotic members Bcl-2, Bcl-xL and Mcl-1. Induction of MOMP requires inhibition of the latter proteins by the so-called BH3-only Bcl-2 family relatives, notably Bid and Bim. Recently, c-Myc has been shown to be an important regulator of apoptosis priming through its ability to promote the expression of the pro-apoptosis Bcl-2 family proteins, Bax, Bid and Bim [23], thereby controlling intrinsic (mitochondrial) apoptosis thresholding. Conditions of stress, which are characteristic of rapidly growing tumours, seem likely to be important for the constitutive apoptosis of aggressive cancers.

**Figure 1.**
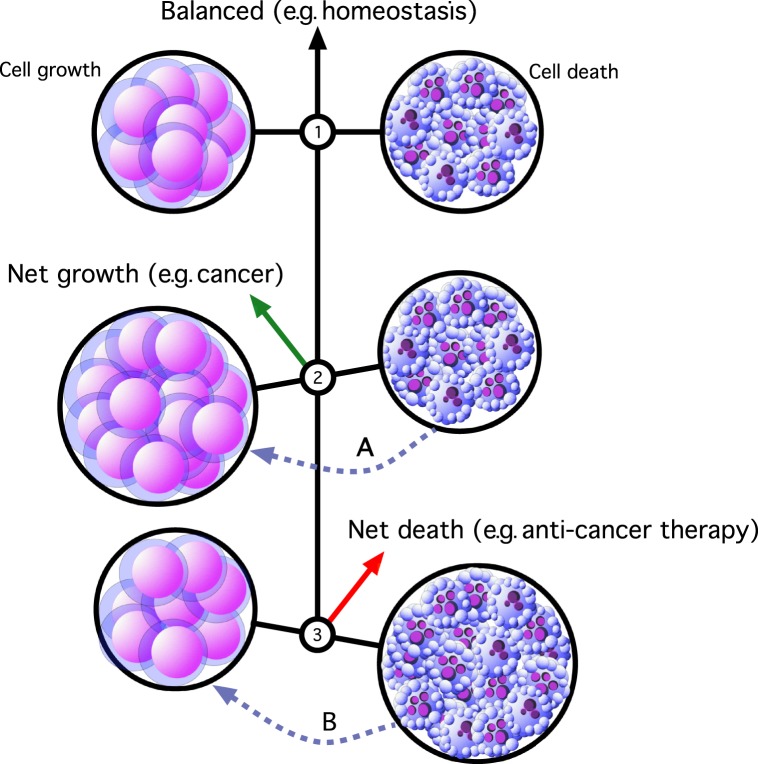
Imbalances in proliferation and death in cell populations of relevance to cancer. (1) Balanced expansion (left) and death (right; here illustrated by apoptosis) of cells within a population—as occurs in homeostasis—results neither in net growth, nor net death, and the population remains at a set size. (2) Imbalance caused by proliferation outpacing apoptosis results in net population expansion (green arrow) as occurs in cancer. Direct or indirect signals from apoptotic cells may feed forward into the population expansion side, for example to promote tumour growth (dashed grey arrow, A). (3) Net reduction of cell populations occurs when apoptosis outpaces proliferation (red arrow), for example as a result of an apoptosis-inducing anti-cancer therapy. Mitogenic signals emanating from apoptotic cells (dashed grey arrow, B) may facilitate relapse. Here we propose that signals A and B form the driving force in a conceptual ‘onco-regenerative niche’.

Therefore, far from being free from cell death, aggressive malignant disease represents an *imbalance* between cell birth and cell death such that the former dominates and net population expansion occurs (figure 1). The objective of therapy is to reverse this balance so that cell deletion is the net result with consequent tumour destruction (figure 1). However, the presence of apoptosis within tumour populations does not simply signify cell loss, for apoptosis offers more than mere cell deletion. Indeed, apoptosis holds important consequences for the tissue in which it occurs, not least in terms of the responses it can engender in its immediate or near vicinity. The capacity of apoptosis to modulate immune and inflammatory responses and to trigger tissue repair and regeneration has important implications for its oncogenic potential.

**Figure 2.**
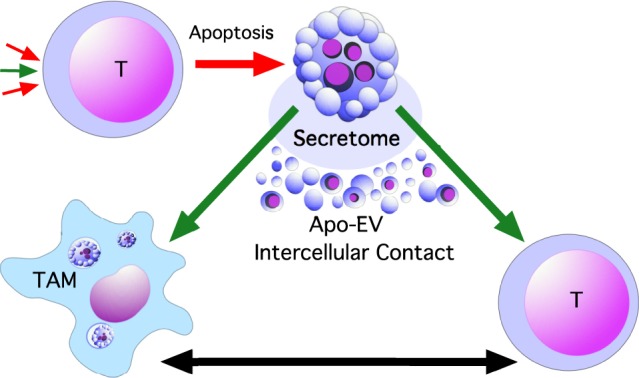
Basic concept of an apoptosis-driven onco-regenerative niche. Apoptosis is induced in tumour cells (T) when pro-apoptosis signalling predominates (e.g. as a consequence of nutrient limitation, anti-tumour immunity or therapy; represented by red arrows, top left). Apoptotic cells generate pro-tumour responses (bold green arrows) in tumour cells and tumour stromal cells such as tumour-associated macrophages (TAM) which also interact with each other (double-headed black arrow). Apoptosis-driven reparatory and immunomodulatory responses of cells in the tumour microenvironment are generated through direct intercellular contact or via release of soluble factors (Secretome) or extracellular vesicles (Apo-EV) from apoptotic cells. It is proposed that the complex network of cells and factors thus generated constitutes the onco-regenerative niche (ORN). The driver of the ORN may be caused by apoptosis of stromal cells as well as tumour cells.

Here the ‘hidden’ pro-tumour properties of apoptosis are considered, both from the perspectives of emerging evidence, and from a speculative standpoint. The concept of our recently proposed, apoptosis-driven ‘onco-regenerative niche’ (ORN) [6] will be developed with particular reference to the roles of apoptosis-responsive tumour-associated macrophages (TAM) and of apoptotic tumour cell–derived extracellular vesicles (Apo-EV) (figure 2).

## 2. Tissue repair responses to apoptosis: phagocytic and anti-inflammatory effects

Classically, apoptosis contrasts with non-programmed, necrotic cell death in failing to incite inflammatory responses and providing tolerogenic signals to the adaptive immune system [24] (although, under certain circumstances, apoptosis can be immunogenic, and necrosis can also be programmed—see [25–27] for recent reviews). In fact, the best-known responses to apoptotic cells are described as anti-inflammatory, characterized by the production of anti-inflammatory mediators, such as TGF-β1, IL-10 and PGE_2_, which are associated with suppression or resolution of inflammatory responses [24,28,29]. The non-phlogistic nature of apoptosis is reflected in the absence of inflammatory effects of the process during normal physiology. For example, in the thymus, an organ in which the vast majority of the cells it produces undergo apoptosis, the process is histologically virtually invisible. This illustrates the rapidity with which apoptotic cells are removed from histological sight and this occurs as a consequence of phagocytosis that is dependent on Rac-1 signalling [13,30]. The phagocytes of apoptotic cells fall into two categories: (i) so-called non-professional phagocytes, neighbouring cells of diverse lineages, including fibroblasts, endothelial cells, epithelial cells and many others that are not otherwise overtly phagocytic, and (ii) professional phagocytes, notably macrophages, be they resident tissue macrophages or macrophages recruited to sites of high-rate apoptosis such as resolving inflammation [13,31–35]. In the latter context, which may occur as a result of tissue damage—with or without infection—macrophage accumulation is an essential part of the inflammatory response that is required for tissue repair. Furthermore, macrophages are key cellular players in anti-inflammatory responses to apoptotic cells although non-professional phagocytes also make important contributions relative to tissue context. Indeed, recent studies have indicated that, in lung inflammation, phagocytic clearance of apoptotic lung epithelial cells is mediated by their viable epithelial neighbours, which are triggered to release anti-inflammatory cytokines via Rac-1-dependent signalling [36]. Intriguingly, polymorphonuclear leucocytes, which form the major cellular arm of the acute inflammatory response to infection or necrosis, are professional phagocytes par excellence, but they are rarely involved in phagocytic clearance of apoptotic cells [37]. Apoptotic cells secrete chemotactic ‘find-me’ signals, including lysophosphatidyl choline [38], sphingosine-1-phosphate (S1P) [39], fractalkine [40], and nucleotides (ATP and UTP) [41], along with ‘keep-out’ signals such as lactoferrin [37,42] in order to ensure selective attraction of mononuclear phagocytes. Hence, both in normal tissues and in tumours, apoptotic cells are readily encountered histologically at sites of apoptosis in association with macrophages.

The phagocytes of apoptotic cells deploy a multitude of receptors in order to engage with, and respond to, apoptotic cells effectively and non-phlogistically. These molecular mechanisms have been reviewed in detail recently and constitute two main categories according to their dependence on phosphatidylserine (PS), a seemingly universal lipid ‘eat-me’ signal that is exposed on apoptotic cells as a result of the activation of a PS scramblase, Xkr8, in the plasma membrane by the effector caspases, caspase-3 and -7 together with the inactivation of flippase activities by the same caspases [43,44]. Phagocyte integral membrane receptors that interact directly with apoptotic cell surface-exposed PS comprise BAI1, TIM4, Stabilin 2, TLT2 and RAGE. Additional, secreted PS receptors that essentially opsonize apoptotic cells include (i) MFG-E8, which bridges to the phagocyte integrins, α_v_β_3_ and α_v_β_5_, and (ii) the Mertk ligands, Gas6 and Protein S (reviewed recently by [35]). Mertk is a receptor tyrosine kinase that is active in tethering and engulfing apoptotic cells and in anti-inflammatory signalling responses of phagocytes toward them [45–47]. It is noteworthy that PS exposure is not sufficient for the phagocytic clearance of apoptotic cells [48]. It is known that further moieties, which, in addition to lipid, include carbohydrate, protein and nucleic acid molecular classes, are exposed at the surface of apoptotic cells. Enzymes that become activated at the plasma membrane are important for the exposure of ligands other than PS that are functional in clearance responses. For example, activation of neuraminidase at apoptotic cell surfaces promotes engulfment [49]. Furthermore, disabled ‘don't-eat-me’ signalling components such as CD31 [50] and CD47 [51] at apoptotic cell surfaces also serve significant roles in facilitating phagocytosis. Indeed, blockade of CD47 suppresses its ability to ligate the inhibitory phagocyte receptor SIRPα and is sufficient to activate engulfment of viable cells independently of PS exposure [52].

The complexity of the pathways available for the engulfment of apoptotic cells may reflect specific axes being needed to engulf specific types of apoptotic cell or those in particular phases of apoptosis—essentially first-line and back-up mechanisms, illustrating the importance of the speed and efficiency of the clearance response. Thus, defects in certain of the clearance pathways—those involving C1q, MFG-E8 and Mertk—cause persistence of apoptotic cells associated with autoimmune, SLE-like disease sequelae [53–55]. By contrast, defects in other pathways such as CD14 and MBL result in persistence of apoptotic cells in the absence of autoimmune disease characteristics [56,57]. These results are consistent with the view that the former pathways signal anti-inflammatory and tolerogenic responses while the latter are mainly involved in tethering of apoptotic cells to phagocytes, their deficiency leaving anti-inflammatory signalling intact. Both in tissue repair and in homeostatic clearance of apoptotic cells, the anti-inflammatory microenvironment prevents unwanted inflammation and autoimmunity. In tissue repair, efficiency in the PS-dependent pathways involving MFG-E8 and Mertk may be essential [58,59].

Phagocytes of different types, including different activation states of macrophages, may also favour deployment of specific receptors for purposes other than phagocytic and anti-inflammatory effects. In this way, specific receptor usage may be coupled to particular tissue responses such as the promotion of cell survival, proliferation, angiogenesis or matrix remodelling, among others. These cellular processes, which are not limited to phagocytes, are likely to be highly important in tissue repair and in regeneration and, as discussed below, may constitute particularly important responses to apoptosis in tumours that promote cancer evolution and progression. Cancer has often been likened to ‘a wound that fails to heal’ [60], bearing many of the hallmarks of chronic inflammation. It is perhaps more accurate to compare cancer to ‘a wound that fails to stop repairing’ [6]. In this analogy, continued apoptosis and the consequent clearance of apoptotic cells in the tumour tissue exemplifies protracted resolution of inflammation. This theme is continued below in consideration of the composition of the ORN.

## 3. Tissue regenerative responses to apoptosis

Extending the viewpoint that apoptosis represents more than mere programmed cell deletion and cell removal, bourgeoning evidence indicates that the apoptosis machinery can also influence tissue regeneration. This makes teleological sense since, according to circumstances, replacement of deleted cells may be required. It should also be noted, by contrast, that apoptosis can also induce further apoptosis in neighbouring cells. This phenomenon of ‘apoptosis-induced apoptosis’ (AiA) was first defined during development of the fruit fly, *Drosophila melanogaster*, and was subsequently found to be responsible for the synchronous cell death of hair follicle cells during catagen in mice. In both cases, AiA was mediated by TNF produced by the apoptotic cells [61].

Regenerative responses associated with apoptosis, often referred to as ‘apoptosis-induced proliferation’ (AiP) or ‘compensatory proliferation’, have been described in various models from mammals to lowly multicellular animals like *Hydra* and planarian flatworms. A striking example is the regenerative response which follows wounding of *Hydra*, a freshwater metazoan which is anatomically simple, essentially comprising a tubular body at one end of which is a foot specialized for attachment to its substratum and at the other extremity a head structure specialized for feeding and excretion. Transverse bisection of this animal in the midline of the body results in both head and foot regeneration. Head regeneration requires the Wnt-β-catenin pathway and is initiated by caspase activation and Wnt3 release from apoptotic interstitial cells. This causes nuclear translocation of β-catenin, synchronous proliferation of interstitial cells and upregulation of Wnt3 in epithelial cells, culminating in head regeneration. Notably, apoptosis is necessary and sufficient to produce the Wnt3 required for head regeneration following midline amputation and apoptosis is even sufficient to induce ectopic head regeneration [62].

The zone of proliferating cells adjacent to the apoptotic zone in regenerating *Hydra* is reminiscent of the proliferating zones characteristic of the blastemas of regenerating limbs (although the role of apoptosis or caspase activation in establishing these has not been defined, cf [63]) and of the compensatory proliferation that is observed associated with apoptosis in *Drosophila* imaginal discs. In *Drosophila*, the compensatory proliferation that occurs in imaginal disc compartments neighbouring those in which apoptosis is induced, is also regulated via Wnt-β-catenin, as well as BMP and Hh, signalling [64–67]. Conserved signalling pathways associated with regeneration coupled to apoptosis have been observed in mammalian systems too. Hh signalling, for example, has been shown to link apoptosis of hepatocytes with compensatory proliferation of liver progenitors and myofibroblasts [68]. Liver regeneration, along with skin wound healing, has been found to be dependent optimally on the activity of the effector caspases, caspase-3 and -7. Intriguingly, downstream regenerative responses to effector caspase activity led to production of PGE_2_, which, in addition to its modulatory effects on inflammation, promotes progenitor cell proliferation [69] and activates Wnt signalling [70]. PGE_2_ has also been reported to promote tumour repopulation (post irradiation) and angiogenesis downstream of caspase-3 activation and apoptosis in murine tumour models including xenografts of human tumour lines [15,71]. These studies point to PGE_2_ as a common pro-regeneration and pro-tumour mediator produced by apoptotic cells—both transformed tumour cells and tumour stromal cells—as a consequence of effector caspase activation. However, since PGE_2_ is also a well-known anti-inflammatory mediator produced by macrophages that engulf apoptotic cells [29], it is probable that TAM also contribute to this mechanism. Intracellular signalling molecules in cells undergoing apoptosis that are associated with their proliferation-inducing activity in various animal models include PKCδ, Akt, MAPK and JNK [63,66,72–74]; in *Drosophila*, diverse additional pathways have also been implicated, including p53, Notch, EGF and Hippo (reviewed in [75]).

## 4. The concept of the onco-regenerative niche

The importance of tissue niches, otherwise known as microenvironments, is well known in regeneration and cancer. Paget's ‘seed and soil’ hypothesis, initially concerned with the mechanisms surrounding the ability of certain tumour cells (the ‘seed’) to metastasize to certain tissues (the ‘soil’) [76], provides a stimulating conceptual foundation for the evolution of knowledge of tumour microenvironments, their association with tissue repair and regeneration and their significance in post-therapeutic relapse. We recently proposed the concept of the ‘onco-regenerative niche’ (ORN) [6], suggesting that cell death in tumours provides a central driving force in shaping the tumour microenvironment by orchestrating a series of interconnected cellular responses and extracellular modifications that promote tumour growth, progression and relapse. The ORN, its constituent key cells, molecules and activities, is a ‘skeletal’ concept at present, serving as a framework for improving our understanding of the broad implications of cell death in tumours, be it constitutive as part of cancer pathogenesis, or induced as a consequence of therapy. The association of apoptosis and apoptotic cell clearance with wound healing, tissue regeneration and aggressive malignant disease provides the initial basis for developing the ORN concept. Here, TAM (that are activated by apoptotic cells) and extracellular vesicles (EV, that are produced by apoptotic cells), are proposed as two important ORN components (figure 2).

## 5. Roles of macrophages

Although it is well known that the macrophage is a constituent of the innate immune system and has anti-tumour capability, in most (though by no means all) tumours, the macrophage component tends to support the growth of established tumours and facilitates their progression and metastasis [6,77–79]. The tumour-supportive activity of TAM stems from signals arising from the tumour that effectively polarize the functional status of the macrophages towards a reparatory phenotype, often referred to as ‘M2-like’, which starkly contrasts with the M1 or M1-like phenotype of macrophages that mediate anti-tumour effector functions. The tumour-supportive properties of TAM, which have established roles in promoting cell growth, angiogenesis and matrix remodelling, while at the same time suppressing anti-tumour immune responses, are reminiscent of the properties of macrophages that are driven by their interaction with apoptotic cells [6,13]. In fact, apoptotic cells are drivers of M2-like activation of macrophages [80] and impose dominant effects on M1 macrophages, potently suppressing their anti-tumour activity [81]. The ‘M1-suppressive’ property of apoptosis has serious implications for tumour repopulation post therapy and suggests that the very apoptosis of tumour cells that is triggered by anti-tumour, M1-like macrophages could lead to repolarization of the latter to become M2-like, tumour supportive cells. This is a particularly important consideration if TAM repolarization from pro- to anti-tumour is to be useful as an effective anti-tumour immune therapy.

**Figure 3.**
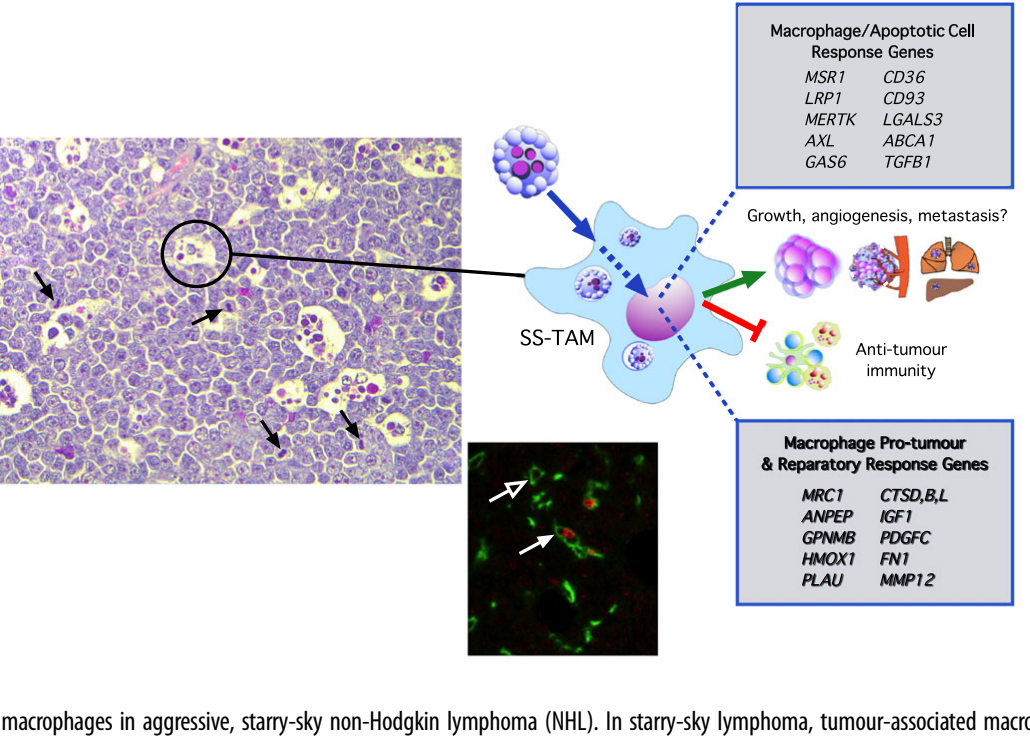
Pro-tumour macrophages in aggressive, starry-sky non-Hodgkin lymphoma (NHL). In starry-sky lymphoma, tumour-associated macrophages (SS-TAM, e.g. circled left) are observed actively engaged in the clearance of apoptotic cells. Note also the high rate of proliferation in these tumours as illustrated by the frequent mitotic figures (black arrows). *In situ* transcriptomics of SS-TAM shows that they upregulate numerous genes involved in responses to apoptotic cells and in pro-tumour and reparatory responses, providing strong evidence that apoptosis may drive numerous pro-tumour macrophage activities such as promoting cell growth, angiogenesis, matrix degradation and metastasis while at the same time inhibiting anti-tumour immunity. SS-TAM are capable of proliferating *in situ* as shown by the filled white arrow (lower centre panel). This shows an example of a mouse macrophage in a starry sky human NHL xenograft co-labelled for macrophage F4/80 (green fluorescence) and the proliferation marker, Ki67 (red). Non-proliferating macrophages are also present (e.g. open white arrow). Faint red signal in the background is present in the nuclei of proliferating human lymphoma cells. For further information, see reference [14].

Recent work from our laboratory has shown that the accumulation and activation of macrophages in aggressive, B-cell non-Hodgkin lymphoma (NHL) is controlled by constitutive apoptosis in the tumour cells [14]. In B-cell NHL that display a ‘starry-sky’ histological appearance (figure 3) the ‘stars’ representing the accumulating TAM, set in a ‘sky’ of tumour cells, prototypically the tumour architecture of Burkitt's lymphoma, BL), the frequent TAM are clearly engaged in interaction with numerous apoptotic tumour cells that are generated constitutively in these rapidly growing tumours. In BL xenografts, TAM accumulation was inhibited by suppression of apoptosis by Bcl-2 or Bcl-xL and tumour growth and angiogenesis were constrained in parallel. Gene expression profiling of the TAM *in situ* clearly demonstrated their potential for supporting tumour growth, angiogenesis, matrix degradation and for suppressing anti-tumour immunity (figure 3). Profiled starry-sky TAM also highly expressed genes associated with known apoptotic cell clearance pathways, such as *LRP1*, *MERTK*, *AXL* (a close relative of *MERTK*), *GAS6, CD36* and *LGALS3* (the gene encoding galectin-3). Studies of the relative significance of these pathways in supporting tumour growth are in progress. Notably, galectin-3 deficiency profoundly inhibits tumour growth in a murine syngeneic transplantation model of aggressive starry-sky NHL [81].

The *in situ* global gene expression profile of starry-sky TAM illustrates the likelihood that multiple tumour-supportive signalling pathways are activated in these cells as a consequence of interaction with apoptotic cells in the tumour microenvironment, particularly apoptotic tumour cells but possibly apoptotic stromal cells too. Little is yet known about the cross-talk between the various ligand-receptor pathways involved in the interactions between macrophages and apoptotic cells and their relevance to the ORN, although it is notable that find-me signals can promote engulfment. For example, fractalkine (CX_3_CL1) stimulates MFG-E8 production and enhances apoptotic cell clearance [82], while S1P activates a novel erythropoietin-mediated phagocytic and tolerogenic pathway [83]. These observations illustrate a potentially important principle—that mononuclear phagocytes may become pre-programmed to activate specific phagocytic, anti-inflammatory and possible additional responses in preparation for key functional activities in the locale of the apoptotic cell. This may be important in harnessing particular phagocyte responses during recruitment to the ORN. Furthermore, opsonins of apoptotic cells and anti-inflammatory mediators produced either in response to apoptosis, or directly by apoptotic cells may act to facilitate mononuclear phagocyte accumulation and reparative activity. Thus, the lung surfactant protein, SP-A acts as a chemoattractant for alveolar macrophages and promotes their engulfment of apoptotic cells [84,85]. Furthermore, TGF-β, which is produced by macrophages as a consequence of apoptotic cell clearance, or even directly by apoptotic cells themselves [24,86], activates chemotaxis of monocytes, stimulating their ability to induce fibroblast growth [87]. Similarly, IL-10—which can also be produced in response to apoptotic cells or again by apoptotic cells themselves [28,88]—promotes both apoptotic cell clearance and survival, thereby facilitating growth of malignant B cells [89]. As we have demonstrated recently, apoptosis promotes macrophage accumulation in NHL, which seems likely to result from a combination of recruitment and proliferation *in situ* [14] (figure 3). However, the extent to which apoptotic cell-activated resident tissue macrophages and apoptotic cell-recruited macrophages deploy common or differential signalling pathways in response to apoptosis has not been defined.

## 6. Roles of apoptotic cell–derived extracellular vesicles

Intercellular communication pathways provide critical networks in the tumour microenvironment including, conceptually, the ORN. Apoptotic cells communicate with their neighbours, whether phagocytic or non-phagocytic, (i) by direct cell-to-cell contact, (ii) by release of bioactive molecules into the extracellular milieu (the apoptotic ‘secretome’), and (iii) through production of membrane-bound sub-cellular vesicles, including apoptotic bodies (figure 2). Apoptotic bodies are difficult to define and the term has been used in various descriptions, ranging from the remains of the dead cell body to the ‘blebs’ that are released from the dying cell. Often, apoptotic bodies are arbitrarily separated from other EV released from apoptotic cells (hereafter referred to as Apo-EV) on the basis of size, with Apo-EV being less than 1 micron and apoptotic bodies ranging from 1 to several microns in diameter [90]. However it is well accepted that size is unreliable in functionally defining different EV types and EV heterogeneity remains a most important challenge in EV biology. Cargoes associated with populations of Apo-EV and apoptotic bodies have been described and, like other EVs, include integral plasma membrane proteins, enzyme systems and RNAs, with apoptotic bodies also harbouring organelles and nuclear components including DNA and histones [91–94]. Therefore, subcellular vesicles produced by apoptosis in the tumour microenvironment offer much potential to modulate the ORN.

Significantly, Apo-EV from endothelial cells have been shown to be capable of immunomodulatory activity [93]. Furthermore, apoptotic bodies are known to be able to transfer genomic DNA, including oncogenes, to phagocytes, although current knowledge indicates that gene expression arising from such transfer is only short-lived because of p53-dependent DNA damage responses in recipient cells [95–97]. Since mutant oncogenes characteristic of tumour cells can also be found in neighbouring tumour stromal cells [98], it seems plausible that horizontal gene transfer may occur via apoptosis in the ORN, thereby providing a novel route to transient or long-lived stromal cell activation. Whether or not this is the case, further studies are warranted to determine the extent to which apoptotic cell–derived vesicles drive tumour growth and progression, especially since EV from non-apoptotic sources have proven capacity in oncogenic activities as well as in tissue repair and regeneration [99–101].

## 7. Conclusion and future perspectives

Apoptosis, fundamentally important in physiological and therapeutic anti-tumour activities, can also engender pro-tumour effects through its impact on tumour cells, immune cells and stromal cells of the tumour microenvironment. It seems likely that the apoptosis programme will also prove to be able to operate systemically. Its pro-tumour properties are closely tied into the tissue repair and regenerative responses that are dysregulated in malignant disease but the underlying molecular mechanisms are, as yet, only poorly understood. Given the indications that pathway usage in translating apoptosis to compensatory proliferation is evolutionarily conserved from *Hydra* to mammals, we hypothesize that conserved tissue repair and regenerative molecular pathways driven by cell death are hijacked in diverse cancer types. There are many challenges in testing this hypothesis, and future research will need to address several fundamental questions. Critically, how are responses to apoptosis differentially regulated? For example, what are the signals that permit discrimination between apoptosis-induced proliferation, AiP, and null responses to apoptosis (no further loss and no replacement), which may also, in some cases at least, include apoptosis-induced survival of neighbours [102]? What are the relative roles of molecular interactions achieved via intercellular contact or transfer through the secretome of apoptotic cells or via apoptotic EV? What is the relative importance of apoptosis of tumour cells versus apoptosis of stromal cells and immune cells, including cancer-associated fibroblasts, mesenchymal stromal cells and TAM? To what extent does this differ between tumour types? In order to define clear roles for apoptosis, it will be crucial to discriminate between cell stress (i.e. molecular mechanisms that can induce apoptosis) and apoptosis *per se*. It will also be important to discriminate mechanisms that specifically require apoptosis from those that are dependent on non-apoptotic roles of the apoptosis machinery such as caspases, which are known to perform diverse non-apoptotic functions. Finally, do other manifestations of regulated cell death such as necroptosis, autophagy and ferroptosis contribute to the ORN?

It is our hope that the concept of the ORN will provide a platform from which to answer these and other important questions concerning the ways in which cell death can impinge on the tumour microenvironment. Because both constitutive and therapy-induced cell death processes are so fundamental in cancer biology and treatment, knowledge gains in this area hold much promise for future developments in improving the long-term treatment of a broad range of cancers.

## Data accessibility

This article has no additional data.
